# An Insight Into Tuberculosis Patients in the Chest Clinic of North India: Epidemiological Profile and Treatment Outcomes in the Wake of COVID-19

**DOI:** 10.7759/cureus.47161

**Published:** 2023-10-16

**Authors:** Saran Singh, Shweta Gupta, Abhinav Jha, Deepak Dhamnetiya, Ravi P Jha

**Affiliations:** 1 Department of Respiratory Medicine, Dr. Baba Saheb Ambedkar Medical College and Hospital, Delhi, IND; 2 Department of Medicine, Dr. Baba Saheb Ambedkar Medical College and Hospital, Delhi, IND; 3 Community Medicine, Atal Bihari Vajpayee Institute of Medical Sciences and Dr. Ram Manohar Lohia Hospital, New Delhi, IND; 4 Department of Community Medicine, Dr. Baba Saheb Ambedkar Medical College and Hospital, Delhi, IND

**Keywords:** tb-hiv co-infection, mdr-tb, post-covid era, treatment outcome, tuberculosis

## Abstract

Objectives

Our study aims to re-evaluate the epidemiological profile and treatment outcomes of TB patients enrolled at the chest clinic of a tertiary care center after the third wave of COVID-19 in New Delhi.

Patients and methods

We have conducted an observational analytical study after taking the IEC approval from October 2022 to February 2022 on the TB patients enrolled from March 2022 to August 2022. The total data of 1114 TB patients was analyzed. The association between various factors and treatment outcomes was assessed using the chi-square test. To identify the independent effects of these factors on treatment outcomes, we did a multiple logistic regression analysis.

Results

We found that the treatment outcomes were mostly successful (83.9%, n=935), while a few patients lost to follow-up (11.7%, n=130) and died (4.4%, n=49). Deaths were significantly higher among geriatrics (19%, n=15), PTB (4.9%, n=30), and MDR TB (15%, n=3). The treatment success was highest among the new category of patients (85.1%, n=807), followed by retreatment patients (80.1%, n=117) and MDR TB patients (55%, n=11). Adults and geriatrics had a significantly higher risk of death (4.45 times and 27.93 times, respectively) compared to pediatrics. In addition, death risks were higher among males (1.6 times for females), MDR TB patients (17 times for new patients), and HIV-reactive patients (3.05 times for HIV non-reactive patients).

Conclusion

We found that males, HIV-TB co-infection, the geriatric population, pulmonary TB patients, and MDR TB were at a higher risk of death. By identifying high-risk groups, policymakers can prioritize targeted interventions and allocate resources effectively to address the specific needs of these vulnerable populations.

## Introduction

Tuberculosis (TB) is an infectious disease that mainly infects the lungs, known as pulmonary TB, but can also affect other parts of the body, such as the lymph nodes, abdominal organs, bones and spine, meninges, etc., known as extra-pulmonary TB [1]. The TB burden accounts for 10.6 million cases and 1.6 million deaths (reported in 2021 as per the Global Tuberculosis Report, 2022). South East Asia Region of WHO continues to bear the brunt of the TB burden, accounting for 45% of global TB cases. The highest burden of TB is among adult men (57%), compared to adult women (33%) and children (11%). [2]. In India, there was an increment of 19% of the total number of TB patients in 2021 (compared to 2020), accounting for 1.9 million TB cases. In India, the incidence of TB was 188 per 100,000, and the mortality rate was 37 per 100,000 in 2020. The rising trend in TB cases in India can be attributed to improved TB surveillance and reporting mechanisms [3]. Delhi, India's capital, reported the highest prevalence of 747 cases per 100,000 people [4]. In India (year 2018), the treatment success rates of TB for drug-sensitive and drug-resistant cases were 81% and 48%, respectively [5].

The COVID-19 pandemic has caused an evident reduction in healthcare services for TB, such as active surveillance and treatment facilities. The World Health Organization (WHO) reported an 18% reduction in the notification of TB cases in 2020 (compared to 2019). WHO and the Stop TB Partnership have predicted 11.4 million additional deaths related to TB (from 2020 to 2025). In India, the vaccination of BCG was reduced by 1 million in April 2020 (compared to April 2019). The National Institute for Communicable Diseases (NICD), South Africa, reported reductions in testing and case detection of TB, synchronous with peaks of COVID-19. The increased incidence of TB and decreased treatment completion rates could also have increased due to higher contact saturation because of lockdowns, worsened poverty, a reduction in HIV/type 2 diabetes control, a reduction in the BCG vaccination rate, decreased access to TB healthcare facilities; TB medication supply chain delays, etc. [6,7].

Overall, TB is a complex disease that requires a multi-sectorial collaborative approach to reduce the burden of TB. There is a need to increase efforts to tackle the social determinants of TB, identify undiagnosed cases, and improve treatment outcomes through highly effective interventions [3]. The latest data has brought to light concerns regarding existing perceptions about TB and health programs. However, there is limited literature available on the evaluation of the epidemiological profile and treatment outcomes of TB patients in Delhi post-COVID duration. While some studies have been conducted in the past, it is crucial to re-evaluate them on a real-time basis, reflecting the effects of COVID-19. Therefore, this study aims to re-evaluate the epidemiological profile and treatment outcomes of TB patients enrolled at the chest clinic of a tertiary care center after the third wave (last wave) of COVID-19 in New Delhi.

## Materials and methods

**Figure 1:**
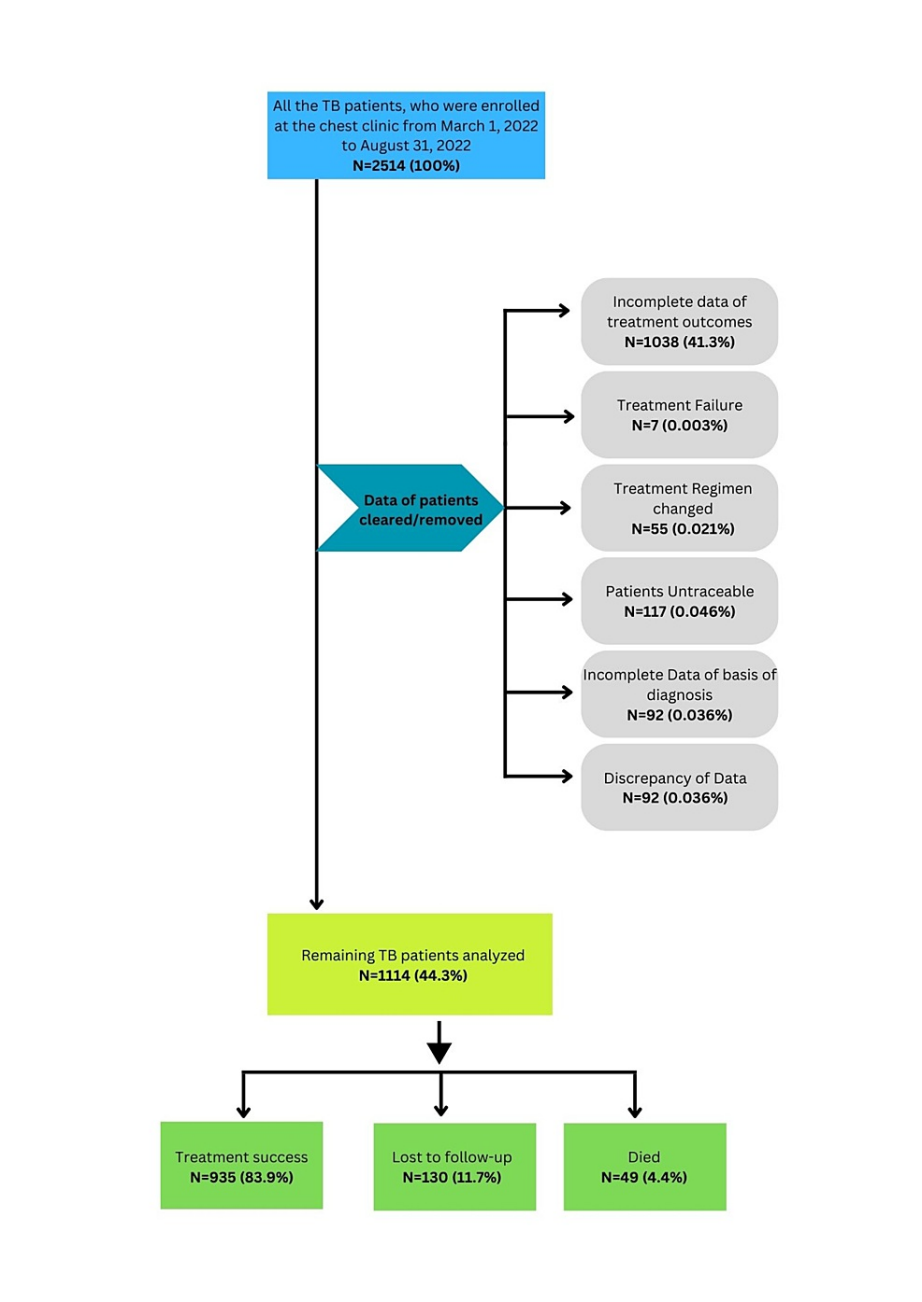
Flowchart for the selection of participants N= Number of subjects, %= Percentage of subjects

We have conducted an observational record-based study from October 2022 to February 2023 on the TB patients enrolled from March 2022 to August 2022 at the chest clinic of a tertiary care hospital in New Delhi, India, after getting the necessary approval from the Institutional Ethical Committee (5(32)/2020/BSAH/DNB/PF 22606-07). The Chest Clinic consists of a total of 15 TB units/DOTS Center and covers a population of 1 million. The required sample size was determined using the formula, with an assumed average proportion of 69.2% of PTB patients among the total TB patients at the DOTS center [6]. Considering a 95% level of significance, a 5% relative error, and a design effect of 1.5, the minimum sample size was 1026. The investigators went to collect data themselves to reduce the risk of information bias. We included all TB patients enrolled at the chest clinic between March and August 2022. Participants with incomplete or discrepant data were excluded. Ultimately, the study included 1114 participants (Figure 1). To ensure quality assurance, various activities were undertaken, including checking participant eligibility, ensuring data completeness, and preventing double data entry.

Data collection

The data for this study was collected from the chest clinic. The data included socio-demographic information, a unique identity number of the patient (NIKSHAY ID), smear results, diagnostic investigations, details of treatment, HIV testing results, diabetes-related information, and substance use-related information. The NIKSHAY ID of all enrolled patients was obtained from the data storage system in the chest clinic. The investigators extracted data for each patient enrolled at the chest clinic from the NIKSHAY website (https://www.nikshay.in/).

Data management and statistical analysis

A Microsoft Excel sheet (Redmond, USA) was used to record the data. Categorical data was analyzed using percentages and proportions. Quantitative data was summarized using the mean and standard deviation. IBM Corp. Released 2012. IBM SPSS Statistics for Windows, Version 21.0. Armonk, NY: IBM Corp. was used for data analysis. The association between the epidemiological profile, HIV-TB coinfection, and diabetes comorbidity and treatment outcome was assessed using the chi-square test. To identify the independent effects of these factors on TB treatment outcomes, we conducted the analysis using a multinomial logistic regression model. A box plot is used to represent the variation in age with respect to treatment outcomes. A significance level of p < 0.05 was considered statistically significant.

We have used World Health Organization (WHO) criteria to categorize TB patients as per treatment outcome status [8]. For the analysis, we focused on three main categories: treatment success, patients lost to follow-up, and those who died. Treatment success was considered a reference.

## Results

We conducted an analysis on a total of 1114 TB patients registered between March 1st 2022, and August 31st 2022. Most TB patients, about 74% (n=824), belonged to the adult age group, while pediatric TB patients accounted for 18.9% (n=211) of the total number of TB patients. Among the patients, 52.6% (n=586) were males, while the remaining were females. Pulmonary TB patients accounted for 55.9% (n=618), while the rest were classified as having extrapulmonary TB. Among the extrapulmonary TB cases, 30.1% (n=147) of the cases had lymph node TB, 28.1% (n=137) had abdominal TB, and 24% (n=117) had pleural TB. Most of the patients were newly diagnosed TB cases (85.1%, n=948), and only 1.8% were identified as multi-drug resistant (MDR) TB patients. Among the 1114 patients, 3.9% (n=44) were reactive for HIV, and out of the 1038 TB patients, 9.7% (n=108) were diagnosed with diabetes. A total of 60.3% (n=672) of the TB patients were clinically diagnosed. The treatment success rate was found to be 83.9% (n=935); 11.7% (n=130) of patients were lost to follow-up, and 4.4% (n=49) died (Table 1).

**Table 1:** Distribution of TB patients according to socio-demographic and clinical characteristics n= Number of subjects; %= Percentage of subjects; SD= Standard deviation Other*: Eye, ear, skin, nose, oral cavity, larynx, tongue

Variable (n=1114)	Frequency (%)/Mean ± SD
Age (in completed years)	31.35 ± 16.00
Pediatric (0-18)	211 (18.9)
Adult (19-60)	824 (74.0)
Geriatric (above 60)	79 (7.1)
Sex	
Male	586 (52.6)
Female	528 (47.4)
Weight (in Kgs) (n=1072)	45.07 ± 14.11
Classification (n=1006)	
Pulmonary TB	618 (55.9)
Extra-pulmonary TB	488 (44.1)
Site of extra-pulmonary TB (n=488)	
Lymph node	147 (30.1)
Abdominal	137 (28.1)
Pleural	117 (24.0)
Bone (excluding spine)	11 (2.3)
Spinal	8 (1.6)
Meninges	7 (1.4)
Genitourinary	4 (0.8)
Miliary	4 (0.8)
Pericardial	1 (0.2)
Other^*^	52 (10.7)
Category of the patient	
New	948 (85.1)
Retreatment	146 (13.1)
MDR TB	20 (1.8)
HIV status	
Reactive	44 (3.9)
Non-reactive	1002 (89.9)
Unknown	68 (6.1)
Diabetes mellitus status (n=1038)	
Diabetic	108 (9.7)
Non-diabetic	871 (78.2)
Unknown	59 (5.3)
Basis of diagnosis	
Clinically diagnosed TB	672 (60.3)
CBNAAT	160 (14.4)
Sputum microscopy	264 (23.7)
Sputum culture	18 (1.6)
Microbiologically positive	
Yes	442 (39.7)
No	672 (60.3)
Treatment outcome status	
Treatment success	935 (83.9)
Lost to follow-up	130 (11.7)
Died	49 (4.4)

We found that treatment success was highest among the pediatric age group (90.5%), followed by adults (84.2%), and least among geriatrics and the elderly (63.3%). Deaths were significantly higher in geriatrics (19%) as compared to adults (3.8%) and pediatrics (1.4%) age groups. We found that treatment success was higher among females compared to males. Loss to follow-up and deaths were significantly higher in males than females. The treatment success rates were higher among extra-pulmonary TB patients (compared to pulmonary TB), and deaths were significantly higher among PTB (4.9%) as compared to EPTB (2.3%). Among EPTB cases, the highest treatment success was reported in TB with lymph node involvement, and significantly higher deaths were reported in other (24.5%) groups of EPTB sites. The treatment success was highest among the new category of patients (85.1%), followed by retreatment patients (80.1%), and least among MDR TB patients (55%). Deaths were significantly higher among MDR TB (15%) patients as compared to new (3.8%) or retreatment (6.8%) category patients. Significantly higher deaths were reported in patients with unknown HIV (25%) or unknown diabetic status (10.2). Statistically significant higher deaths were reported among microbiologically positive (6.1%) patients as compared to microbiologically negative TB patients (3.3%) (Table 2).

**Table 2:** Association of TB treatment outcome with socio-demographic and clinical characteristics among study subjects Values mentioned: n (%); n= Number of subjects, %= Percentage of subjects #p-value: considered significant if p-value < 0.05 *Others: Bone, spine, genitourinary, miliary, pericardial, meninges, eye, ear, skin, nose, oral cavity, larynx, tongue

Variables	Treatment success	Lost to follow up	Died	p-value^#^
Age (in completed years)				
Pediatric (0-18)	191 (90.5)	17 (8.1)	3 (1.4)	0.000
Adult (19-60)	694 (84.2)	99 (12.0)	31 (3.8)	
Geriatric (above 60)	50 (63.3)	14 (17.7)	15 (19.0)	
Sex				
Male	470 (80.2)	84 (14.3)	32 (5.5)	0.002
Female	465 (88.1)	46 (8.7)	17 (3.2)	
Classification (n=1006)				
Pulmonary TB	507 (82.0)	81 (13.1)	30 (4.9)	0.017
Extra-pulmonary TB	428 (87.7)	49 (10.0)	11 (2.3)	
Site of extra-pulmonary TB (n=488)				
Lymph Node	137 (93.2)	7 (4.8)	3 (2.0)	0.039
Abdominal	119 (86.9)	16 (11.7)	2 (1.5)	
Pleural	102 (87.2)	14 (12.0)	1 (0.9)	
Other	70 (16.4)	5 (45.5)	12 (24.5)	
Category of the patient				
New	807 (85.1)	105 (11.1)	36 (3.8)	0.002
Retreatment	117 (80.1)	19 (13.0)	10 (6.8)	
MDR TB	11 (55.0)	6 (30.0)	3 (15.0)	
HIV Status				
Reactive	34 (77.3)	7 (15.9)	3 (6.8)	0.000
Non-reactive	875 (87.3)	98 (9.8)	29 (2.9)	
Unknown	26 (38.2)	25 (36.8)	17 (25.0)	
Diabetes mellitus status (n=1038)				
Diabetic	90 (83.3)	15 (13.9)	3 (2.8)	0.005
Non-diabetic	765 (87.8)	83 (9.5)	23 (2.6)	
Unknown	44 (74.6)	9 (15.3)	6 (10.2)	
Basis of diagnosis				
Clinically diagnosed TB	582 (86.6)	68 (10.1)	22 (3.3)	0.040
CBNAAT	128 (80.0)	19 (11.9)	13 (8.1)	
Sputum microscopy	211 (79.9)	40 (15.2)	13 (4.9)	
Sputum culture	14 (77.8)	3 (16.7)	1 (5.6)	
Microbiologically positive				
Yes	353 (79.9)	62 (14.0)	27 (6.1)	0.007
No	582 (86.6)	68 (10.1)	22 (3.3)	

Our analysis revealed that geriatric TB patients had a significantly higher risk (27.93 times) of death compared to the pediatric population. Males had a 1.6-times higher risk of death compared to females. Patients with MDR-TB had a significantly high risk of death, with a 17-fold higher risk compared to new patients. Additionally, MDR patients had a 4.3 times higher risk of being lost to follow-up. Among the TB patients, those who were HIV reactive had a 3.05 times higher risk of death and a 1.65 times higher risk of being lost to follow-up compared to HIV non-reactive patients (Table 3).

**Table 3:** Multiple logistic regression analysis of TB treatment outcome with socio-demographic and clinical characteristics among study subjects O.R.: Odds ratio, C.I.: Confidence interval

Variables	Treatment outcome	
	Lost to follow up	Died
	OR (95%C.I.)	OR (95%C.I.)
Age (in years)		
Pediatric (0-17)	1 (Reference)	1 (Reference)
Adult (18-59)	1.793 (0.917 - 3.506)	4.445 (0.899 - 21.978)
Geriatric (≥60)	3.439 (1.366 - 8.658)	27.938 (4.824 - 161.807)
Sex		
Female	1 (Reference)	1 (Reference)
Male	1.510 (0.979 - 2.329)	1.606(0.722 - 3.573)
Site of disease		
Pulmonary TB	1 (Reference)	1 (Reference)
Extra Pulmonary TB	0.904 (0.505 - 1.618)	0.739 (0.273 - 1.999)
Category of the patient		
New	1 (Reference)	1 (Reference)
Retreatment	0.914 (0.489 - 1.710)	2.326 (0.903 - 5.990)
MDR TB	4.348 (1.475 - 12.820)	17.073 (3.682 - 79.162)
HIV		
Non-Reactive	1 (Reference)	1 (Reference)
Unknown	7.366 (1.634 - 33.210)	1.435 (0.082 - 25.103)
Reactive	1.649 (0.581 - 4.674)	3.049 (0.458 - 20.282)
Diabetes Mellitus		
Non-Diabetic	1 (Reference)	1 (Reference)
Unknown	2.276 (0.960 - 5.399)	8.139 (2.470 - 26.818)
Diabetic	1.049 (0.503 - 2.188)	0.463 (0.101 - 2.113)
Microbiologically Positive		
No	1 (Reference)	1 (Reference)
Yes	1.420 (0.799 - 2.524)	0.670 (0.246 - 1.826)

**Figure 2:**
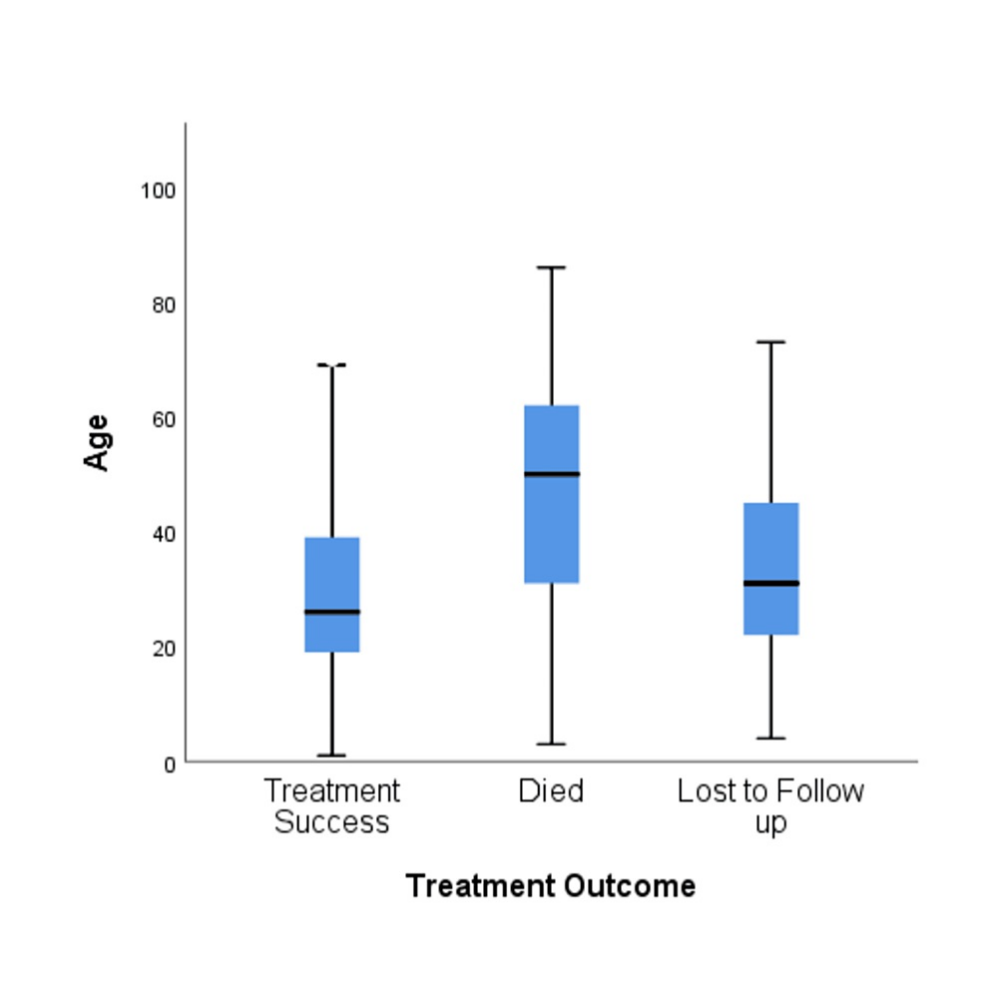
Box plot depicting variation in age with respect to treatment outcome

The mean age of dead patients was higher as compared to treatment success/loss to follow-up categories (Figure 2).

## Discussion

In our study, we found that most of the patients enrolled were successfully treated, while 4.4% (n=49) of the patients died. It is noteworthy that a significant proportion of the deceased individuals belonged to the geriatric age group, which corroborated findings from a similar study conducted by Murali et al. in South India [9]. This finding is similar to the pre-COVID time. The higher deaths among the elderly population can potentially be attributed to factors such as compromised immune systems, inadequate patient care, underlying health conditions, co-morbidities, limited social support, difficulties in understanding and complying with treatment, as well as delays in seeking appropriate healthcare facilities [10,11]. 

In contrast to a different study [12], our findings indicate that males face a nearly 1.5 times higher risk of mortality. We observed a higher risk of death among pulmonary TB patients vis-à-vis extra-pulmonary TB patients, which differs from the conclusions drawn by a few other studies [13,14]. One possible explanation for this discrepancy is the involvement of the respiratory system, which compromises the vital process of oxygenation in patients. Among the different forms of extra-pulmonary TB, TB lymph node patients exhibited the most favorable treatment outcomes, possibly due to the presence of apparent symptoms, early diagnosis, and fewer complications [15]. 

MDR TB patients had a treatment success rate of 55% (n=11), and the risk of death was 17 times higher compared to newly diagnosed patients. This increased risk could potentially be attributed to factors such as increased virulence of the MDR TB strain, longer treatment duration, expensive drugs, ineffective drug regimens, delayed diagnosis, and outdated treatment policies [16]. We found that TB patients co-infected with HIV had nearly three times the risk of death and a lower treatment success rate compared to HIV non-reactive patients, corroborated by several other studies [17,18]. This can be explained by the compromised immune state of the patients due to HIV infection, delayed presentation and diagnosis of HIV, poor adherence to both anti-retroviral therapy (ART) and anti-tubercular treatment (ATT), as well as the presence of other co-existing conditions like neoplasms and secondary infections [17]. Smear-negative patients had a higher risk of mortality compared to smear-positive patients, which could be due to the uncertainty of TB infection and, hence, inappropriate/ineffective treatment. 

In 2020, during the months of April and May, coinciding with the lockdown due to the COVID-19 wave, there was a 63% decline in TB case notifications (compared to 2019), while in 2021, the decline was 52%. This decline could potentially be attributed to the absence of active surveillance as well as the stigma and discriminatory behavior that emerged in our society following the pandemic. These factors may have led individuals to hide their TB symptoms and refrain from seeking any healthcare facility because of stringent quarantine measures [16]. A study has shown an increased risk of TB among individuals who recovered from COVID. This increased risk might be due to the immune-suppressive nature of COVID, the medications administered for COVID-19 treatment, such as steroids, as well as the presence of underlying co-morbidities in those patients [3]. In the year 2019, before the COVID-19 pandemic, the treatment success rate and death rate were 82% and 4%, respectively [19]. Whereas we found in 2022 that the treatment success rate and death rate were 83.9% (n=935) and 4.4% (n=49), respectively, showing a slight increase. 

There are a few limitations to consider in our study. First, the data we used had missing information. Consequently, our results may have been either overestimated or underestimated. Second, our study was conducted as a retrospective, single-centered study, which limits the generalizability of our findings. We believe that our findings still provide valuable insights. Nevertheless, further research incorporating comprehensive data collection methods and broader study settings would be beneficial for a more robust understanding of the topic.

## Conclusions

Our study found that most TB patients achieved successful treatment outcomes. However, certain subgroups, including males, individuals with HIV-TB co-infection, the geriatric population, pulmonary TB patients, and those with MDR TB, were at a higher risk of death. These results have important implications, particularly in the post-COVID era, as they provide policymakers with up-to-date data to inform appropriate measures for the control of TB. By identifying high-risk groups, policymakers can prioritize targeted interventions and allocate resources effectively to address the specific needs of these vulnerable populations.
